# Management of sexual partners of pregnant women with syphilis in northeastern Brazil – a qualitative study

**DOI:** 10.1186/s12913-019-3910-y

**Published:** 2019-01-24

**Authors:** Ana Fátima Braga Rocha, Maria Alix Leite Araújo, Angélica Espinosa Miranda, Rodolfo Gómez Ponce de Leon, Geraldo Bezerra da Silva Junior, Lea Dias Pimentel Gomes Vasconcelos

**Affiliations:** 1 University of Fortaleza-UNIFOR. Av. Washington Soares, 1321, Edson Queiroz, Fortaleza, Ceará CEP 60.811-905 Brazil; 2 Department of Infectious Diseases – Federal University of Espírito Santo. Av. Marechal Campos, 1468, Maruípe, Vitória, Espírito Santo, CEP 29040-091 Brazil; 3 Latin American Center for Perinatology/Women and Reproductive Health (CLAP/WR), Pan American Health Organization (PAHO – WHO), Av Brasil 2679, Montevideo, Uruguay; 4 Fortaleza Health Secretariat, Rua do Rosário, 263, Centro, Fortaleza, Ceará CEP: 60.055-090 Brazil

**Keywords:** Congenital syphilis, Syphilis in pregnancy, Male-partner involvement, Health services

## Abstract

**Background:**

Although there are public policies for eradicating congenital syphilis, they do not seem to be a routine in most health services. The objective of this study was to evaluate the management of sexual partners of pregnant women with syphilis in primary health care in northeastern Brazil.

**Methods:**

This is a qualitative assessment carried out from February to October 2014 in the city of Fortaleza, Ceará, northeastern region of Brazil, through the observation of six primary health care centers and interviews with 21 professionals, six coordinators, nine women diagnosed with syphilis during antenatal care and four sexual partners. The data were submitted to thematic content analysis.

**Results:**

Important flaws were identified at the primary health centers studied regarding the management of syphilis during pregnancy. Accessing testing and treatment is difficult, and there are no standardized strategies to notify the partner. The responsibility for notifying them is transferred to the women, and counseling does not offer proper guidance nor sufficient emotional support to help them.

**Conclusion:**

The management of pregnant women and their sexual partners in our region does not comply with global recommendations. Professional qualification, sensitization, and standardization of health professionals’ conduct are necessary. Offering support to health professionals on their clinical practices by means of a supervision process may contribute to the adoption of the recommended guidelines and to the promotion of care based on privacy, respect, confidentiality of information, and awareness of the problems faced by women as a result of syphilis diagnosis.

**Electronic supplementary material:**

The online version of this article (10.1186/s12913-019-3910-y) contains supplementary material, which is available to authorized users.

## Background

The reduction in the incidence rate of Congenital Syphilis (CS) to less than 0.5 cases per 1000 live births and its elimination as a public health problem in Latin American and Caribbean countries are goals of national [1] and international [2, 3] health organizations. However, CS still remains a challenge for poor and developing countries such as Argentina, Paraguay, and Brazil, which still faced high incidence rates, 1.21, 2.90, and 6.49, respectively, in 2015 [4], mainly due to the poor quality of antenatal care [4–6].

In Brazil, national policy guides that pregnant women should be tested for syphilis at the first antenatal visit [7]. The test should be repeated during the third trimester of pregnancy and at delivery. Seropositive women and their sexual partners should be treated [7]. The non-treatment of sexual partners is one of the main factors hindering the control of CS [8] and remains a challenge for health professionals. Official statistics [9] show that between 1998 and June 2016 only 12.7% of the partners of syphilis seropositive pregnant women were treated. This fact emphasizes the importance of efforts to improve on timely and adequate treatment for pregnant women and their partners.

Brazil’s Ministry of Health (MOH) states that the notification of sexual partners of persons with Sexually Transmitted Infections (STIs), including syphilis in pregnancy, should involve different strategies, which goes from the notification of an index patient to active search of this patient’s partner [10]. These strategies can certainly help health professionals, but it is necessary to consider that the diagnosis of an STI brings to light some delicate situations such as the possibility of unfaithfulness, fear of communicating the diagnosis due to the risk of suffering violence, and fear of breaking off the relationship [11–13]. All these facts can be considered barriers that difficult the treatment of the partners of syphilis-seropositive pregnant women.

Despite scientific evidence that contacting sexual partners of persons with STIs is difficult [14], it is believed that it is possible to locate partners of pregnant women with syphilis, as these women often live with the fathers of their babies and maintain a link with the primary health care centers [15, 16]. Therefore, treatment should take place at this level of care [17]. However, there is a history of invisibility of men in these services [18], which are unattractive from the point of view of men and seen as distinctly feminine spaces [19, 20].

In Brazil, some attempts have been made to attract men to health services – for instance, the implementation of the National Comprehensive Healthcare Policy for Men (*Política Nacional de Atenção Integral à Saúde do Homem*) [21]. However, in order to achieve a better coverage of treatment to sexual partners of pregnant women with syphilis, it is necessary to properly train professionals to meet the subjective demands that arise from diagnosis.

Given this context, the present study aims to evaluate the management [notification, testing, treatment and follow-up] of sexual partners of pregnant women with syphilis in primary health care in a metropolitan area of northeastern region of Brazil.

## Methods

A qualitative assessment was carried out aiming to contribute to elucidate the aspects relating to the low proportion of partners of pregnant women with syphilis treated at primary health care centers (PHC).

In Brazil, health care is provided to the population by its national health system, the *Sistema Único de Saúde* (SUS). It is designed in a hierarchized network whose services are provided in different levels of complexity; primary, secondary, and tertiary care. Primary care is the front door to SUS and it works according to the Family Health Strategy (FHS) program whose teams include physicians, nurses, nurse assistants, and community health agents (CHA).

The FHS teams are responsible for a geographically circumscribed area and should attend to 4000 people at most. This makes it possible to provide a follow-up care and establish a link of responsibility between professionals and patients [22]. It is worth highlighting the fact that only 53.4% of Brazilian families were attended to by FHS teams in 2013 [23].

The study was conducted in the city of Fortaleza, capital of the State of Ceará, located in northeastern region of Brazil. The city has a population of 2.5 million inhabitants and, as far as health care is concerned, is divided into six Health Coordination Offices (HCO), whose function is to enforce sectoral policies, establish specific goals for each population group and provide articulated services within a social protection network.

The present research was conducted in six PHCs, one from each HCO, whose selection criterion was the number of notifications of syphilis in pregnant women in 2013. In Brazil, despite syphilis in pregnancy being subject to compulsory notification since 2005, there is a considerable sub-notification of cases. Fortaleza has 110 PHCs and the six centers participating in this research have notified 27.7% of all notified cases in the municipality.

Data were collected from February through October 2014 by means of observation and semi-structured interviews that were made simultaneously. Researchers stayed 45 days on average at each center.

As to observation, a script was applied comprising the availability of HFS teams, materials and the necessary inputs for managing pregnant women and their partners: Rapid Test for Syphilis, the Venereal Disease Research Laboratory (VDRL) test, benzathine penicillin G, syringe, needles, distilled water, educational material such as flyers, posters, information leaflets, World Health Organization (WHO)/Pan American Health Organization (PAHO)/Ministry of Health (MOH) protocols, publication and manuals. The data were registered on the field notebook.

In an attempt to understand how the management of the sexual partners was done, the perceptions of the different social actors involved in this context were examined. Therefore, physicians, nurses, PHC coordinators, women diagnosed with syphilis during antenatal care and their sexual partners were interviewed.

Twenty-one health care professionals participated in this research (seven physicians and 14 nurses) out of a total of 43 (20 physicians and 23 nurses) who made up the FHS teams of the selected centers. The interviews were held at PHCs at previously scheduled times. Twelve professionals (11 physicians and one nurse) refused to participate due to lack of time for the interview and ten could not participate because they were either on vacation or on work leave during the period of data collection. Every PHC has a coordinator responsible for the center’s management and all of them accepted to participate in the research.

In the year 2013, 19 pregnant women with syphilis were notified at the evaluated centers. With the help of the health centers, they were contacted by phone and informed about the research objectives. Then, they were invited to attend the center for interviews. All of them attended the center and nine were included, as they were the only ones who had had the same partner since the time of diagnosis.

In order to address the theme more naturally, establish a connection, and help the women feel more comfortable expressing themselves, the interviewers initially asked about the antenatal care and exams done during such period. The application of this strategy resulted in the women telling about the diagnosis of syphilis and made it possible to bring up the subject in a more welcoming atmosphere.

At the time of the interviews, the interviewer asked each woman about her relationship with her partner, the revelation of the diagnosis of syphilis, and her opinion on her partner participating in the research. Those who admitted to the possibility of their partners’ participation were given the researcher’s contact number and seven days’ time for them to talk with their partners and call back to confirm it or not. Five partners declined the invitation and four accepted to participate if the interviews were carried out at home – a condition that was accepted.

Contact with the sexual partners was done by the women out of respect for ethic matters and due to the possibility of not revealing the diagnosis. All precautions were taken to prevent these women from finding themselves in any embarrassing situation. Furthermore, it was desirable to preserve the women’s right of having the partner participate or not in case they believed they could go through any constraint, violence, or bring harm to their relationship.

All interviews were conducted by a nurse (woman) vastly experienced on researching and qualified to deal with sexually transmitted infections. The researcher composing the team of authors has never worked at any of the assessed health care centers nor has ever had contact with the interviewees previously to the present study.

The interview questions addressed the process of notification, testing, treatment and follow-up of pregnant women with syphilis and their sexual partners. The interview guide was developed and pre-tested for this study (Additional file 1). Each interview lasted for about an hour on average and were recorded after obtaining consent from the participants.

Collected data were submitted to thematic content analysis to identify the core meanings of the interviews [24, 25]. All the interviews were transcribed verbatim and revised by the first author to ensure the accuracy of the transcripts. In the pre-analysis phase, the transcripts were read by the first four authors so that they could become familiar with the data. In the second phase, the material was explored, and the results were treated. Similar sub-themes were grouped into broad themes and organized into categories by the first author and revised by the other three authors. In the phase of interpretation of data, the assessment of the management of the partners of women with syphilis was carried out in compliance with recommendations of the WHO/PAHO/MOH [1, 3, 10, 26].

All participants were informed about the research and, then, were asked to sign an Informed Consent Form and were free to give up their participation at any moment. The interviews were always conducted individually and in places where privacy and confidentiality could be preserved. Participants were identified according to the order in which they were interviewed and to their professional category in order to ensure anonymity.

## Results

Out of the 21 interviewed professionals, 19 were women, 14 had a degree in Nursing and seven in Medicine. Age varied between 25 and 60, most participants were aged between 35 and 50 (12). Graduate time ranged from six months to 31 years and most of them had been graduated for more than five years (16). Working time at the health care center was under three years for nine professionals.

Four coordinators had a degree in Dentistry and two in Nursing. They were aged between 33 and 60 and their professional working time ranged from three to 29 years. As coordinators working at this health care center, their working time varied from one to three years.

The interviewed women aged between 18 and 33, had less than eight complete years of schooling, and seven of them were unemployed. Four women had a history of illicit drug abuse, one of them had already been sent to jail, and seven others had gone through the full treatment for syphilis.

Concerning the four sexual partners, three were aged over 30 and had between five and eight complete years of schooling. All of them were employed, two had a history of illicit drug abuse, three had already been sent to prison, and two had gone through the full treatment for syphilis.

Having the interviewees’ statements as a starting point and based on the recommendations for the notification, testing, treatment and follow-up of sexual partners of pregnant women with syphilis, institutional weaknesses that jeopardize the management of the sexual partners were identified and organized into three thematic categories: Lack of knowledge about and non-adherence to strategies for partner notification “Poor access to testing and gaps in counseling” and “Obstacles to the testing and treatment in primary care”.

### Lack of knowledge about strategies for partner notification

The physicians and nurses stated that they did not feel prepared to adequately manage the sexual partners of pregnant women with syphilis. They revealed that after entering the family health strategy teams, they received training on how to assist pregnant women. However, the themes developed during training courses focused on the progression of pregnancy and antenatal care and did not approach partner notification or dealing with situations resulting from revealing the diagnosis of syphilis.

Moreover, none of the centers provided manuals and protocols for supporting professionals and clarifying their doubts. Also, there were no educational materials in use providing guidelines and clarifications to the patients.

*I always try to clear up my doubts over the internet. The educational material is restricted to the regional office. You need to request it in advance, schedule a day; it is not inside the consulting room or accessible to the patient, so we cannot show the pictures. It would be ideal. (Professional 04, nurse).*

The professionals also declared that they were unaware of the existence of MoH protocols and guidelines which provide steps to be followed for notification. It is possible that this might be one of the reasons why the health centers do not adhere to a schematic strategy for partner notification. Notification was limited to health professionals telling the pregnant woman that her partner needs to attend the center for treatment without providing any alternatives in case she has difficulties telling her partner about it.

*As a manager, I see that it depends a lot on the professional, because there are professionals who notify and schedule a day for the pregnant woman to bring her husband in and there are those who ask the pregnant woman to impart information on the treatment. (Coordinator 05, nurse).*

*We have no strategies for notification, we just say it, talk to the patient, provide information on the risks and the need for treating her and her partner. (Professional 01, physician).*

Professional conduct varies among professionals at the same center – some of them provide treatment prescriptions for the patient to take to her partner.

*The physicians usually write the prescription and ask the pregnant woman to take it to her partner. (Professional 04, nurse).*

When asked about their conduct towards partner’s non-attendance, the health professionals stated that they were unable to take any actions other than continuing the pregnant woman’s care and did not show any discomfort or reported any need to change this current practice. There were suggestions to get the CHA involved in the active search for those partners. However, they reported on being concerned about ethical issues and the possibility of breaching the confidentiality of diagnosis.

*There is nothing to do other than carrying out the pregnant woman’s consultation if the partner does not attend the center. (Professional 12, physician).*

*It would be good if we could count on the CHA, but they would have to be properly trained in confidentiality issues. Today I am afraid of that because I do not know if everyone will know about it the next week. (Professional 11, nurse).*

### Poor access to testing and gaps in counseling

The centers had professionals trained to perform rapid tests and these tests were available at all centers. However, none of the centers carried out these tests during antenatal consultations. The VDRL was the test used to diagnose syphilis and all physicians and nurses were unaware of the need to carry out monthly serological tests to follow-up pregnant women with syphilis and their partners.

The women and their partners reported on difficulties accessing the test and also a delay in the delivery of results. It was observed that none of the centers collected biological material on a daily basis, which hindered the access to diagnosis. This situation forced people to return to the centers several times or to adopt other strategies for testing.

*I could take the tests at the center, they even told me that, but I preferred to pay for taking them otherwise it would take too long (Women 06).*

*I went to the center to get a blood test but it was not possible. They scheduled a day. So, I went there on the scheduled day and they did not do it. They said they were not doing it that day and that I should go there another day. I cannot miss work, so I did not go anymore. (Partner 02).*

*We are not carrying out rapid tests during antenatal consultations because these consultations would take too long and we’d get overloaded with work. So, until we get to organize ourselves, we’ll pick one specific day of the week to perform rapid tests and will keep on running the VDRL only. (Professional 03, nurse).*

Taking the women’s statements as a starting point, it was possible to verify that when they came for the VDRL test results, the orientation they got from counseling was restricted to informing on the test results and the need for treating their sexual partners and left aside the couple’s relationship context.

Possibly out of fear of how their partners would react, or insecurity in explaining about their diagnosis, the women seemed to feel uncomfortable about talking to their partners. They highlighted the importance of receiving support and orientation from professionals to help them go through this situation. Some even mentioned they looked for help on the Internet.

*I froze up when I tried to talk to him, I was shaking. I went up to him and said I had something serious to talk about. Then he got worried, asked about the name of the disease and a lot of things I could not explain. Then we searched the Internet. (Women 04).*

*I think women are supposed to tell their partners, but the professionals should help us by telling us how we should talk to them because it is very difficult. He [the partner] might even think I am making it up. (Woman 01).*

When asked about the disclosure of the diagnosis, the partners said they had been informed by the pregnant woman about the need for treatment only. They reported that the women did not know how to provide any additional information about the problem.

*She could not quite explain it to me, she just showed me some papers she had brought saying we should get some injections. (Partner 03).*

### Obstacles for treatment in primary health care

The professionals were reluctant to accept treating pregnant women and their partners at the primary care center claiming there is a risk of anaphylactic reactions. Benzathine penicillin was available in four of the six centers analyzed, but it was administered in only one center. All the other centers provided the medicine to the pregnant woman and advised her to seek other levels of care. It was observed that all the centers had needles, syringes and distilled water.

*The medication is not administered here because we still do not have enough trained personnel to provide urgent care. Therefore, the professionals do not feel confident to do so. We have the medication, but we provide it to the patient and ask them to take it in a hospital setting. (Coordinator 04, dentist).*

Additionally, there was certain discomfort among the few professionals who advocated the administration of penicillin. They demonstrated fear of generating conflicts between colleagues.

*I’m really in favor of carrying out treatment in the primary care level, but sometimes, even at the same center, there are difficulties. Many colleagues don’t feel secure to administer penicillin and, therefore, are totally against it. So, I end up falling into their routine of not carrying out treatment to avoid conflict. (Professional 13, physician).*

Inability to provide treatment at point of diagnosis means that the pregnant woman and her partner move to a facility that provides a different level of care. Therefore, they need to spend money on transportation or, in the absence of financial resources, walk long distances. This practice adds a new barrier to treatment access.

*To tell you the truth, it (the other health facility) is very far, but there was no other way, we (the couple) went there on foot. (Woman 07).*

*I’m not going to lie, we didn’t go down to the hospital for treatment. They (health professionals) have to understand that we can’t afford it. We can’t afford the bus fare! (Partner 04).*

The professionals recognize that the non-treatment of the woman and her partner in primary care hinders the follow-up and control of the doses administered.

*If the treatment were carried out here, it would be a lot easier, because you would be sure that he is actually taking the medication properly and you could monitor him. (Professional 17, nurse).*

When asked about the treatment administration, the women and their partner stated that they face many administrative drawbacks or barriers when they need to take the medication at a health service facility that provides a different level of care. These services are overcrowded and do not always recognize the validity of the prescription given by the primary care provider, requiring the pregnant woman and her partner to have a new consultation to get a prescription with a stamp bearing the name of the physician on call. In many situations, they even refuse to administer the medication on the grounds that it is the duty of primary care providers.

*Every time we went there, we had to wait for a new consultation. We waited our turn just to get a stamp bearing the name of the physician on call. And it took us a long time [nervous] to get to talk to the physician. I really had to raise my voice inside the hospital in order to get it. After realizing all the difficulties, I decided to pay to take the medication, but drugstores were not administering it anymore. So, we had to go back to the hospital to take the medication there again. (Partner 01).*

*The worst of it is that some hospitals are already sending patients back because they know there is an executive order of the health secretariat stating that health centers must administer penicillin. (Professional 17, nurse).*

There is also the discomfort and exposure to situations of embarrassment as the woman and her partner need to disclose the diagnosis again to another professional with whom they have no bonds. In addition, this new professional does not even recognize them or know their life histories and providing one-time care only.

*It was terrible for me because there are all kinds of services there and I had to go through all that embarrassment. I had to tell what I had again just because I needed to get an injection. That was not necessary at all… sometimes we [the couple] got there at seven o’clock in the evening and arrived home at one o’clock in the morning. Once I even asked at the health center why I could not get it there, and they told me it was because there were no professionals able to administer it. That is an absurd! (Woman 8).*

It was also observed that the primary health care centers analyzed still lack structural conditions and organization of the work process to meet the recommendations for the adequate management of the sexual partners of pregnant women with syphilis.

There was a shortage of human resources, materials and inputs. Some family health teams were incomplete, mainly due to the absence of a physician. In addition, many areas were left uncovered by the family health care strategy due to a shortage of teams or the number of incomplete teams, which jeopardizes access, follow-up, and the establishment of a bond with the partners.

*This health center covers only around 40% of the territory, that is, 60% of the population is not covered. (Coordinator 06, dentist).*

*My main difficulty [in treating the partner] occurs when the pregnant woman is not from the coverage area. They initiate antenatal care but we are not able to track the partner, we cannot monitor him. (Professional 08, nurse).*

A summary of the different positions of the coordinator, health professionals, pregnant women and their sexual partners can be seen in Table 1.

**Table 1:** Management of sexual partners of pregnant women with syphilis in primary care: Different positions of the interviewees

Themes	Coordinators	Health Professionals	Pregnant Women	Sexual Partners
Partner Notification	Left the partner notification strategy for health professionals	Did not feel prepared to notify the partner Did not adopt the strategies recommended by the MoH Held women accountable for partner notification	Felt insecure to notify the partner Considered professional help to disclose the diagnosis was important	Difficulty in understanding the information passed on by the pregnant woman and the importance of attending the center
Testing	Difficulty in implementing the rapid test during antenatal care Difficulty in collecting biological material for VDRL on a daily basis	They did not perform the rapid test during antenatal consultations because doing so would extend the duration of the consultation	Difficulties in being tested and in receiving VDRL test results	
			Did not perform serological tests to follow-up	
Treatment	Do not implement syphilis treatment in the center for fear of anaphylactic reaction	Did not deliver treatment in the center for fear of anaphylactic reaction Missed follow-up and control of the doses administered	Attended more distant centers to receive treatment Long queues for treatment in a center of greater complexity and Exposure to situations of embarrassment	

## Discussion

The present study demonstrated that the analyzed centers, in a region of northeastern of Brazil, did not adhere to any of the strategies recommended by the MOH for partner notification. The centers would only inform the pregnant women about the importance of treating their partners. As was found by a study carried out in Bolivia [11], also in South America, the present study verified that women do not feel secure to tell their partners about the diagnosis of syphilis. Therefore, the responsibility to convince the partner to attend the service should not be transferred to pregnant women only, for they are more vulnerable to suffering gender-based violence [12]. Women also fear that their relationship may end and are concerned about being blamed for the infection [27, 28].

It was also identified that some centers provided drug prescriptions to the woman to take to her partner. Despite being frequently used in some countries in cases of urethral discharge [29], patient-delivered prescription is not considered the most appropriate strategy in the case of syphilis because the drug should be injected, and this should be done in a health care facility. In addition, health care providers miss the opportunity to provide the partner with information on the consequences of non-treatment of syphilis and possible repercussions for the baby.

There is no monitoring of sexual partners by the centers and the professionals are worried about the involvement of the CHA in this process. In Rio de Janeiro, Brazil, a previous study showed that such worry is mainly related to the fact that the CHA lives in the same area as the patient they serve and have personal relationships with them, which could compromise the uptake and confidentiality of patient’s information [30]. It should be noted that the CHA is a member of the Family Health Strategy team and difficulties in dealing with issues arising from STI diagnosis are not specific to this category [31]. The concern for confidentiality within primary care may be related to the fact that men with STI’s prefer to be treated at private centers or drugstores far from their home, as shown in other studies [32, 33].

It has been suggested, nonetheless, that primary care is the best place to treat pregnant women with syphilis and their sexual partners. A study carried out in Peru showed that pregnant women with syphilis who received antenatal care were more likely to have their sexual partners treated [34], reinforcing the idea that the connection with primary care is important for the administration of the partner’s treatment. However, in order to carry it out with no constraints, all PHC professionals need to receive training to improve confidentiality and ethical care practices/attitudes.

On the other hand, primary health care in Brazil is undergoing a high professional turnover [35] that, associated with excess demand, work overload, and limited consultation time [36], hampers the counseling, care, and follow-up provided to pregnant women with syphilis and their sexual partners.

Quality care for pregnant women with syphilis goes beyond the simple prescription of treatment. It involves preventing the transmission to the baby – as well as future infections – and breaking the chain of transmission, fundamental conditions which require addressing sensitive issues that are often difficult for professionals [31] and patients. The provision of education materials can help patients to understand the information provided by health care professionals considering that the women and partners interviewed had low levels of education.

Pregnant women with syphilis need to be provided with counseling and emotional support to help them reveal the diagnosis to the partner. In such an occasion, health professionals must take into consideration the cultural aspects that could have influence on possible reactions from partners [11–13]. This support must be done by means of qualified listening, which can foster their participation in identifying the best notification strategies.

One strategy that can minimize the discomfort generated by the diagnosis of syphilis is the inclusion of the partner in antenatal care [37]. This strategy removes the need to request partner attendance at the health care center following diagnosis of the pregnant woman. Thus, the rapid test can result in the early diagnosis [1] and immediate treatment of the couple.

This studied verified that some of the partners attended health units; however, they faced difficulties accessing testing and treatment. This item of data leads to the reflection that despite the cultural aspects interfering with the use of primary health care services by men [18], the concern with the baby’s health can be a favorable aspect for the partner to attend the health service for treatment, situation verified by a study carried out in Tshwane, South Africa [38].

Health professionals’ refusal to administer benzathine penicillin at the primary care level is a major obstacle to the treatment of pregnant women with syphilis and their partners. In order to change this situation, the National Commission on the Incorporation of Technologies in the SUS (*Comissão Nacional de Incorporação de Tecnologias no SUS – CONITEC*) [39] reiterated the recommendation [17] on the use of this medication in primary care for the prevention of CS, a situation that needs to be urgently put into effect as the harms resulting from its non-administration are much more serious than the possibility of anaphylactic reactions [40]. Despite the recent and worrying shortage of benzathine penicillin in Brazil [41], such a crisis had not yet affected the city of Fortaleza at the time of data collection. However, even with the availability of this drug, the treatment does not effectively occur in primary care.

The women and partners interviewed experienced several setbacks to receive treatment at units of a higher level of complexity. Forwarding these people to such units for benzathine penicillin administration only is totally unnecessary. These units deal with emergency cases and are usually overcrowded, which increases the difficulties to treatment altogether.

It is clear that the existence of clinical care protocols does not guarantee that the actions proposed by these documents will be effectively carried out. Especially, when it comes to difficult issues, such as the management of an STI and the treatment of sexual partners, there must be a process of activities follow-up by means of instructive supervision. In Brazil, this should be done at the local level by Municipal Health Departments, which are the governmental bodies responsible for developing the actions recommended by the MOH protocols [1, 10].

The present study has some limitations. The first limitation is related to the non-inclusion of women and partners who have broken up their relationships. It is possible that these people, in case they have not been treated, be more vulnerable to infect new partners and contract other STI’s, which contributes to the maintenance of the chain of transmission and, consequently, the high rates of syphilis. The second limitation is related to the fact that the data were collected in 2014. However, as there have been no substantial changes in public policies to prevent mother-to-child transmission of syphilis, the findings are still relevant.

## Conclusions

The management of pregnant women with syphilis and their sexual partners in Fortaleza, Brazil, does not comply with global recommendations. Flaws in counseling, difficulties to access testing, treatment not carried out at the site of diagnosis, and no follow-up were the main hindrances identified. This shows that the guidelines are little known by health professionals and are not implemented at primary health care centers.

In order to make the eradication of congenital syphilis a reality in Brazil, professional qualification, sensitization, and the standardization of health professionals’ conducts are necessary. Furthermore, primary health care centers must undergo a supervision process offering support for the implementation of the recommended guidelines and for the promotion of care based on privacy, respect, and confidentiality of information.

## Additional file

Additional file 1: Interview Guide, List of interview questions used to guide interview. (DOC 31 kb)

## Abbreviations

CHA: Community Health Agents (*Agente Comunitário de Saúde - ACS)*
CS: Congenital Syphilis
FHS: Family Health Strategy *(Estratégia Saúde da Família – ESF)*
HCO: Health Coordination Offices (*Coordenadorias de Saúde – Cores*)
MOH: Ministry of Health
PAHO: Pan American Health Organization
PHC: primary health care centers (*Unidades de Atenção Primária à Saúde – UAPS*)
STI: Sexually Transmitted Infections
*SUS*: Unified Health System *(Sistema Único de Saúde*)
VDRL: Venereal Disease Research Laboratory
WHO: World Health Organization

## References

[1] Ministry of Health (BR). Transmissão Vertical do HIV e Sífilis: Estratégias para Redução e Eliminação [Vertical Transmission of HIV and Syphilis: Strategies for Reduction and Elimination]. Brasília: Ministry of Health; 2014.

[2] Organización Panamericana de la Salud. Iniciativa regional para la eliminación de la transmisión maternoinfantil de VIH y de la sífilis congénita en América Latina y el Caribe: documento conceptual. Montevideo: CLAP/SMR; 2009.

[3] World Health Organization (2007). The global elimination of congenital syphilis: rationale and strategy for action.

[4] Pan American Health Organization. Elimination of mother-to-child transmission of HIV and syphilis in the Americas. Update 2016. Washington, DC: PAHO; 2016.

[5] Dassah ET, Adu-Sarkodie Y, Mayaud P (2015). Factors associated with failure to screen for syphilis during antenatal care in Ghana: a case control study. BMC Infect Dis.

[6] Domingues RMSM, Leal MC (2016). Incidence of congenital syphilis and factors associated with vertical transmission: data from the birth in Brazil study. Cad Saúde Pública.

[7] Ministry of Health (BR) (2016). Protocols of Primary Care. Women’s Health Care.

[8] Magalhães DMS, Kawaguchi IAL, Dias A, Calderon IMP (2013). Sífilis materna y congénita: un desafío. Cad. Saúde Pública..

[9] Ministry of Health (BR) (2016). Boletim Epidemiológico Sífilis [Epidemiological Bulletin Syphilis].

[10] Ministry of Health (BR) (2015). Clinical Protocol and Therapeutic Guidelines for Integral Care to People with Sexually Transmitted Infections.

[11] Klisch SA, Mamary E, Diaz Olavarrieta C, Garcia SG (2007). Patient-led partner notification for syphilis: strategies used by women accessing antenatal care in urban Bolivia. Soc Sci Med.

[12] Andrade RFV, Araújo MAL, Vieira LJES, Reis CBS, Miranda AE (2015). Intimate partner violence after the diagnosis of sexually transmitted diseases. Rev Saúde Pública.

[13] Adams OP, Carter AO, Redwood-Campbell L (2015). Understanding attitudes, barriers and challenges in a small island nation to disease and partner notification for HIV and other sexually transmitted infections: a qualitative study. BMC Public Health.

[14] Khan MR, Ravelomanana N, Van Damme K, Randrianasolo BS, Ramaniraka V, Ranaivo N (2010). Notifying partners of patient with early syphilis in Madagascar: case-finding effectiveness and public health implications. Tropical Med Int Health.

[15] Campos ALA, Araújo MAL, Melo SP, Gonçalves MLC (2010). Epidemiology of gestational syphilis in Fortaleza, Ceará state, Brazil: an uncontrolled disease. Cad. Saúde Pública..

[16] Campos ALA, Araújo MAL, Melo SP, Andrade RFV, Gonçalves MLC (2012). Syphilis in parturients: aspects related to the sex partner. Rev. Bras. Ginecol. Obstet..

[17] Ministry of Health (BR). Portaria N° 3.161, de 27 de dezembro de 2011. Dispõe sobre a administração da penicilina nas unidades de Atenção Básica à Saúde, no âmbito do Sistema Único de Saúde (SUS) [Provides on the administration of penicillin in the units of Basic Health Care, under the Unified Health System (SUS)]; Diário Oficial da União [Official Diary of the Union], 2011.

[18] Gomes R, Moreira MCN, Nascimento EF, Rebello LEFS, Couto MT, Schraiber LB (2011). Men don't come!. Absence and/or invisibility in primary healthcare services Ciênc saúde coletiva.

[19] Couto MT, Pinheiro TF, Valença O, Machin R, Silva GSN, Gomes R (2010). Men in primary healthcare: discussing (in) visibility based on gender perspectives. Interface (Botucatu).

[20] Gomes R, Nascimento EF, Araújo FC (2007). Why do men use health services less than women? Explanations by men with low versus higher education. Cad Saúde Pública.

[21] Ministry of Health (BR) (2009). National Policy for Integral Attention to Men’s Health.

[22] Ministry of Health (BR) (2012). National Primary Health Care Policy.

[23] Malta DC, Santos MAS, Stopa SR, Vieira JEB, Melo EA, Reis AAC (2016). Family health strategy coverage in Brazil, according to the National Health Survey, 2013. Ciênc. saúde coletiva..

[24] Minayo MCS. The challenge of knowledge: qualitative research in health. 12. ed. São Paulo: Hucitec-Abrasco, 2010.

[25] Bardin L. Análise de conteúdo [Content Analysis]. São Paulo: Edições. 2011;70.

[26] World Health Organization (2011). Methods for surveillance and monitoring of congenital syphilis elimination within existing systems.

[27] Kalichman SC, Mathews C, Kalichman M, Lurie MN, Dewing S (2017). Perceived barriers to partner notification among sexually transmitted infection clinic patients, Cape Town. South Africa Journal of Public Health.

[28] Araújo MAL, Silveira CB (2007). Women’s experiences with sexually transmitted disease diagnosis - STD. Esc Anna Nery.

[29] Hogben M (2007). Partner notification for sexually transmitted diseases. Clin Infect Dis.

[30] Beerenwinkel A, Keusen AL (2014). Family dynamics under the view of the family health strategy professional. Saúde debate.

[31] Domingues RMSM, Lauria LM, Saraceni V, Leal MC (2013). Treatment of syphilis during pregnancy: knowledge, practices and attitudes of health care professionals involved in antenatal care of the unified health system (SUS) in Rio de Janeiro City. Ciênc. saúde coletiva..

[32] Balfe M, Brugha R, Donovan DO, Connell EO, Vaughan D (2010). Triggers of self-conscious emotions in the sexually transmitted infection testing process. BMC Research Notes.

[33] Araújo MAL, Leitão GCM (2005). Access to medical appointments by men with sexually transmitted diseases at a health unit in Fortaleza, Ceará. Brazil Cad Saúde Pública.

[34] García PJ, Williams E, Cárcamo CP, Chiappe M, Holmes KK, Peeling RW (2015). Partner notification among Peruvian pregnant women with syphilis. Sex Transm Dis.

[35] Medeiros CRG, Junqueira AGW, Schwingel G, Carreno I, Jungles LAP, Saldanha OMFL (2010). Nurses and doctors turnover: an impasse in the implementation of the family health strategy. Ciênc saúde coletiva.

[36] Silva NR (2011). Decisive factors relating to workload in a primary healthcare unit. Ciênc. saúde coletiva..

[37] Duarte G (2007). Extension of prenatal care to the partners as a strategy to enhance adhesion to prenatal care and to reduce mother-to-child transmission of infections. Rev Bras Ginecol Obstet.

[38] Koo K, Makin JD, Forsyth BW (2013). Where are the men? Targeting male partners in preventing mother-to-child HIV transmission. AIDS Care.

[39] Comissão Nacional de Incorporação de Tecnologias no SUS (CONITEC) (2015). Benzathine penicillin for prevention of congenital syphilis during pregnancy.

[40] Galvao TF, Silva MT, Serruya SJ, Newman LM, Klausner JD, Pereira MG (2013). Safety of Benzathine penicillin for preventing congenital syphilis: a systematic review. PLoS One.

[41] Ministry of Health (BR). Nota Informativa Conjunta N°109/2015/GAB/SVS/MS, GAB/SCTIE/MS. Orienta a respeito da priorização da penicilina benzatina para sífilis em gestantes e penicilina cristalina para sífilis congênita no país e alternativas para o tratamento da sífilis. [It orientates regarding the prioritization of penicillin benzathine for syphilis in pregnant women and crystalline penicillin for congenital syphilis in the country and alternatives for the treatment of syphilis]. Brasilia: Ministry of Health; 2015.

