# Supplementary material for: Management of sexual partners of pregnant women with syphilis in northeastern Brazil – a qualitative study
Source: BMC Health Serv Res. 2019 Jan 24;19:65. doi: 10.1186/s12913-019-3910-y (PMC6344990; doi:10.1186/s12913-019-3910-y)
Supplement: Supplementary file 1 — Interview Guide, List of interview questions used to guide interview. (DOC 31 kb) [file 12913_2019_3910_MOESM1_ESM.doc]

**Interview Question Guide**

**For coordinators and health professionals:**

Identification data:

- Sex
- Age
- Undergraduate degree
- Length of time working as a professional
- Length of employment at the center

Does this center notify sex partners of pregnant women with syphilis?

Is there any strategy in this center to notify the partner? Talk about the strategy.

What if the partner attends the center, how is care provided?

How does this center deliver syphilis testing and treatment?

What do you think of ESF professionals’ work in the process of notification and treatment of sex partners?

Talk about the main difficulties related to examining, testing and treating sex partners of pregnant women with syphilis.

**For women diagnosed with syphilis during antenatal care:**

Identiication data:

- Age
- Education
- Marital status
- Length of relationship with current partner
- Do you live with your partner?
- Occupation
- Drug use history
- Previous incarceration

Tell me a little about how your antenatal care was.

Você conseguiu fazer todos os exames antes do bebê nascer? Precisou pagar por algum exame? Were you able to get all the tests before the baby was born? Did you have to pay for a test?

Were there any altered test results?

What did the health professional tell you about the problem?

How did you feel getting the diagnosis?

Did you get treated? Where did you get treated?

Did the professional give you any information about your partner’s treatment?

Did you disclose your diagnosis to your partner?

How did you feel at that moment?

Did the professional participate in that process?

Did your partner get treated?

**For sexual partners:**

Identification data:

- Age
- Education
- Marital status
- Length of relationship with current partner
- Do you live with your partner?
- Occupation
- Drug use history
- Previous incarceration

Tell me how did get to know the diagnosis.

Did someone call you to attend the center? How did they call you (notification strategy)?

Did you attend the center?

Did you receive any information about the disease (prevention, treatment, condom use)?

Did you get treated at the center?

Did you get any syphilis tests?

Did you have VDRL testing after treatment?
